# Mediterranean moth diversity is sensitive to increasing temperatures and drought under climate change

**DOI:** 10.1038/s41598-022-18770-z

**Published:** 2022-08-25

**Authors:** Britta Uhl, Mirko Wölfling, Claus Bässler

**Affiliations:** 1 Institute for Ecology, Evolution and Diversity, Conservation Biology, Faculty of Biological Sciences, Goethe University Frankfurt, 60438 Frankfurt am Main, Germany; 2 Bio-advice, 97464 Niederwerrn, Germany; 3 Nationalpark Bayerischer Wald, 94481 Grafenau, Germany

**Keywords:** Biodiversity, Environmental health, Conservation biology, Phenology

## Abstract

Climate change affects ecosystems worldwide and is threatening biodiversity. Insects, as ectotherm organisms, are strongly dependent on the thermal environment. Yet, little is known about the effects of summer heat and drought on insect diversity. In the Mediterranean climate zone, a region strongly affected by climate change, hot summers might have severe effects on insect communities. Especially the larval stage might be sensitive to thermal variation, as larvae—compared to other life stages—cannot avoid hot temperatures and drought by dormancy. Here we ask, whether inter-annual fluctuations in Mediterranean moth diversity can be explained by temperature (T_Larv_) and precipitation during larval development (H_Larv_). To address our question, we analyzed moth communities of a Mediterranean coastal forest during the last 20 years. For species with summer-developing larvae, species richness was significantly negatively correlated with T_Larv_, while the community composition was affected by both, T_Larv_ and H_Larv_. Therefore, summer-developing larvae seem particularly sensitive to climate change, as hot summers might exceed the larval temperature optima and drought reduces food plant quality. Increasing frequency and severity of temperature and drought extremes due to climate change, therefore, might amplify insect decline in the future.

## Introduction

Globally, the temperature increased by approximately 1 °C during the last 170 years^1^. In the Mediterranean region, climate change effects are even more pronounced compared to the global average, with a more rapid temperature increase along with lower precipitation rates^2,3^. Therefore, the Mediterranean area is among those regions most vulnerable to climate change^4^. Subsequent effects include increased fire risk^5^, summer drought^6^, low water levels^7^, and ozone-induced stress^8^. Further, climate change has also detrimental effects on biodiversity. A negative effect on animal diversity has repeatedly been reported, with predicted exacerbated extinction rates. However, studies are biased towards large and conspicuous species such as vertebrates^9^. For insects, which face species losses in several regions across the globe^10^, the effects of climate change are less well understood. Climate change seems to have a considerable impact on insect diversity^11^, yet, we have limited knowledge of the mechanisms of how altered climatic conditions affect insect diversity.

Insects, as poikilothermic organisms, are strongly dependent on their thermal environment. Direct climate change effects on insects might occur when temperature optima are exceeded and humidity optima are fallen below. However, in central European regions, climate change still positively affects insect communities, as they are profiting from warm temperatures during their development^11,12^. Nevertheless, there are hints that the positive effect turns into negative during the warmest summer months, as indicated by insect biomass losses throughout Germany^13^. In the Mediterranean region, with a warmer climate, compared to Central Europe, climate change-induced heat and drought might be even more threatening to insect diversity.

Additionally, indirect climate change effects on insect diversity might occur due to changes in the food plant quality. Drought and ozone stress reduce the plants’ photosynthetic activity^14^, terpenoid emission^15^, and cause physiological changes, such as increased root/shoot ratios and decreased growth^16^. Lower food plant quality can reduce larval survivability and reproduction rates and therefore might reduce insect diversity^17,18^. However, disentangling the relative importance of direct thermal effects and indirect food plant-mediated effects caused by climate change poses a challenge to field studies as these factors occur simultaneously^19^.

**Figure 1:**
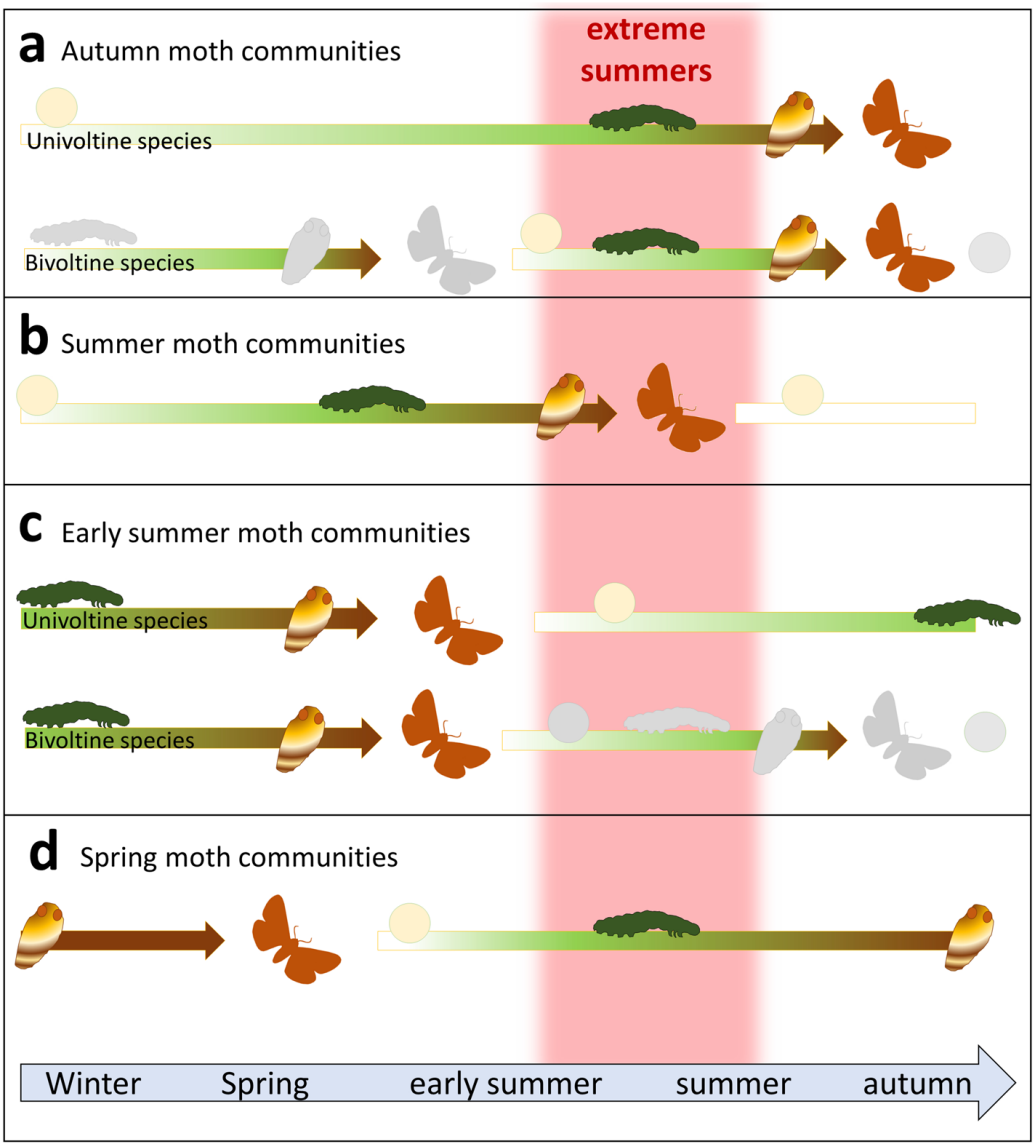
Different lifecycles of moth communities. The temporal occurrence of different life stages is indicated by the color of the arrow (green = larva, white = egg, brown = pupae). Autumn moth communities (**a**) overwinter as egg and then develop as a larva during the summertime. At the end of summer, they pupate and finally hatch as adult in autumn. Summer moth communities (**b**) either overwinter as eggs or as freshly hatched larvae, develop in early summer, pupate, and occur as adult in the summer. Early summer moth communities (**c**) are often overwintering in their larval stage, as praepupae or pupae and—if any—only take up food in spring, then pupate and hatch in early summer as adult moth. The eggs can be found in the summertime. Spring moth communities (**d**) are overwintering as pupae. Adult moths hatch as soon as spring begins. Eggs are laid in the end of spring and then, larvae hatch in the early summer. The main development, therefore, takes place in the summer, similar to the autumn moth communities. For species with bivoltine life cycle (**a** and **c**), the first generation of adults occurs in early summer (with overwintering larvae), while the second generation of adults can be found in autumn and originates from summer-developing larvae. In our analysis, therefore, the first generation was included in the early summer moth community, while the second generation was part of the autumn moth community (for which reason these generations are colored grey in the other season). Hot summers (red area) might have especially strong effects on summer-developing species, as the larval stage is not capable of avoiding unfavorable conditions.

Susceptibility of species richness to direct and indirect effects might depend on the species’ characteristic lifecycle. The diversity of species with summer-developing larvae might be strongly affected, since temperature extremes and reduced food plant quality cannot be avoided by larvae (Fig. 1). The larval stage is the growing phase within the development cycle, in which most food is needed, resulting in rapid weight increase. Larvae are able to hibernate in winter, but they cannot induce summer dormancy, while for all other developmental stages, there are hints that unfavorable conditions (i.e. hot summers) can be avoided by elongated pupal stages, egg- or adult dormancy^20,21^ (Fig. 1). A summer-developing lifestyle, therefore, might be more prone to hot temperatures, compared to a lifestyle, where larvae are overwintering.

Here, we investigate how long-term moth diversity patterns are linked to variation in temperature and precipitation considering differences in life cycle development among species. We expect that inter-annual moth diversity fluctuations can be explained by the climate during larval development. Furthermore, the climate during larval development is expected to have the strongest effect on the diversity of species with summer-developing larvae. We take advantage of a 20-year moth survey and link temperature and precipitation variability across years with species richness and the community composition within a protected area. We address the following hypotheses:

1. Increasing temperatures and decreasing precipitation during larval development have a negative effect on moth species richness. Effects are more pronounced for species with summer-developing larvae.
2. Inter-annual variability of temperature and precipitation during larval development affects the moth community composition. Effects are more pronounced for species with summer-developing larvae.

## Material and methods

### Study area

The study area is a coastal pine forest reserve, located in North-Eastern Italy, near the city of Ravenna (N 44°29′50.82″, E 12°13′49.58″). The Pineta san Vitale reserve (hereafter PsV) consists mainly of a mixed oak-pine forest. Other habitat types such as riparian forests, open grassland, and reed areas also exist. The area developed in the 10th to fifteenth century due to sedimentation processes^22^. Pine forests were soon after planted when the area was under the property of different abbeys^23^. At this time, the forests were used for firewood, pine nut production, and wood pasture^23^. Until the end of the eighteenth century, large areas of the coast were covered by these pine forests, however afterwards, they were cut down until only small forest fragments remained. PsV is one of these fragments, comprising about 950 ha of forest. Today, the forest is protected as part of the Po Delta regional park. Forest management has stopped since the establishment of the regional park in 1988. Since then, natural succession has formed a near-natural forest structure^24^, and only in some parts of the park, horses are used to keep open habitats within the reserve.

### Moth sampling

We surveyed moth communities during the last 20 years, using two trap systems: A manual light tower, equipped with an Osram 500 W HWL lamp, and automated light traps, equipped with two 18 W light tubes (black light and white black light) (Fig. S1). Most of the moth data were collected in the early summer period (end of May–June), comprising samples from 1997 to 2020. For the summer moth communities (end of July-beginning of September) samples from 2002, and then from 2011 to 2020 were available. From springtime (April–May), data from 2011 to 2017 were available, while for autumn moths, data from 2001, and from 2011 to 2014 were gathered. From 2011 to 2017, larger datasets were available^24–26^. All sampling data, including the date of sampling, the locations of the traps (Fig. S2), and which samples were pooled for the analysis can be found in Supplementary Table S1. Although the data structure is quite heterogeneous, our data set allows reliable analyses. Samples within the two trap systems were always sampled in the same way, making them standardized. However, the two systems do have their peculiarities: By using manual light towers, more moths are caught compared to automated light traps^27,28^. Also, the light source and wavelength used can affect the sampled community composition^29^. However, there are hints that these differences are much smaller than previously expected^30^. To account for these trap-specific differences, we always included trap type as a random factor in models (see Data analysis section).

The automated light trap samples were pooled to make sample sizes comparable to the manually gathered ones. This means, that for the 2011 dataset, which was completely sampled by automated light traps, all samples of the same habitat type in one season were pooled^24^. For the 2013 and the 2015–2017 datasets, where only one habitat type was analyzed, automated light trap samples from the same sampling night were pooled. All moths were identified to species level, and genitalia dissection was applied if necessary. The species names were checked following the Fauna Europaea database, to avoid synonyms in the dataset. The complete species list can be found in Supplementary Table S2.

### Climate data

To test our hypotheses, we used temperature and precipitation during larval development. As outlined in the introduction, climatic conditions during the developmental stage are expected to be relevant for moth diversity. For the temperature measurements, we used the archive data of ilmeteo.it (https://www.ilmeteo.it/portale/archivio-meteo) for the region of Ravenna. For precipitation sums, we used data from the surrounding weather stations in Marina di Ravenna, Ravenna, Classe, Fosso Ghiaia, Ponte Chiavica and San Pietro in Vincoli (Supplementary Table S3). We calculated the total precipitation sum (mm) for each weather station and month. The monthly mean sum of precipitation across all weather stations was then used for the calculation of a) annual precipitation (sum of monthly precipitation rates) and b) mean monthly precipitation sum during larval development (mean of the monthly precipitation sum, including all months in which the larvae develop).

We calculated the mean temperature (T_Larv_) and mean sum of precipitation (= humidity, H_Larv_) for larval development times of spring, early summer, summer, and autumn moth communities. The development cycle differs among species, but moths occurring in the same flight period tend to share similar development cycles^31^ (Fig. 1). For spring moths, many species develop in the summer months the year before and enter the pupal stage in autumn. We, therefore, calculated the mean temperature and precipitation during larval development for spring moths, by using the average of the climate measurements from June–September in the year before the sampling. Early summer moths species often have a multivoltine lifecycle. They can occur in early summer with a first generation, and then again in autumn with a second generation. T_Larv_ and H_Larv_ of early summer moths, therefore, were calculated as average from September-May. Summer moths, in contrast, overwinter as eggs or small larva and then develop from April to July (T_Larv_ and H_Larv_ for summer moths). Autumn moth communities were often composed of the second generation of early summer species. The average temperature/precipitation from June to September was hence used as T_Larv_ and H_Larv_ for autumn moths.

### Data analysis

All analyses were performed in the R 4.0.4 environment^32^. We first performed a Mann–Kendall test (package ‘Kendall’^33^) to test if there are trends in the mean annual temperature and precipitation for the time series 1997 to 2020 in the region. The null hypothesis (no trend) was rejected by a *p*-value < 0.05.

To test the first hypothesis, we pooled sampling nights per season and year to obtain robust communities (representing the whole reserve and not just one location). Within one season (equaling one field trip), high sample coverage was always reached, rendering season/year the best unit for species extrapolations. We used individual-based species interpolation-extrapolation for each year/season, to obtain standardized values of species richness^34^. Hence, small samples were extrapolated to double the smallest dataset (108 individuals), while large datasets were rarefied to the same number of individuals. The interpolation-extrapolation graph for species richness as well as the sample coverage is provided in Supplementary Figure S3. We used the standardized values of species richness per season/year as response in linear mixed-effects models with T_Larv_ and H_Larv_ as predictors. The trap type (automated vs. manual light trap) was included as a random factor. Overwintering larvae (early summer species), and larvae developing during one vegetation period (spring, summer, and autumn species) were treated in separate models, as overwintering larvae likely have other temperature optima compared to those developing in summer. For summer 2020, no precipitation rates were available, and we, therefore, excluded the 2020 dataset from the summer larvae model. The outcome of the models was adjusted for multiple comparisons by false discovery rate control^35^. To assure, that the trap type or the different sampling locations did not affect the inferences, we also performed models including only samples from manual traps and only samples from manual traps and from one location (Supplementary table S4). As the results were consistent, we will in the following only interpret the models including all samples.

To test the second hypothesis, we calculated the Sørensen dissimilarity between samples for each season separately, using the package ‘vegan’^36^. We aim to get insights into how communities with overwintering larvae (early summer communities) and communities with summer-developing larvae (spring and autumn communities) respond to temperature and precipitation. We applied permutation tests with 999 permutations (*adonis* function in the ‘vegan’ package) using the distance matrices as response and T_Larv_ as well as H_Larv_ as predictors. Additionally, the trap type was included as a covariate to account for trap type effects. We finally used the Sørensen distance matrix to calculate non-metric multidimensional scaling (*metaMDS* function in the ‘vegan’ package), to visualize community responses.

## Results

**Figure 2:**
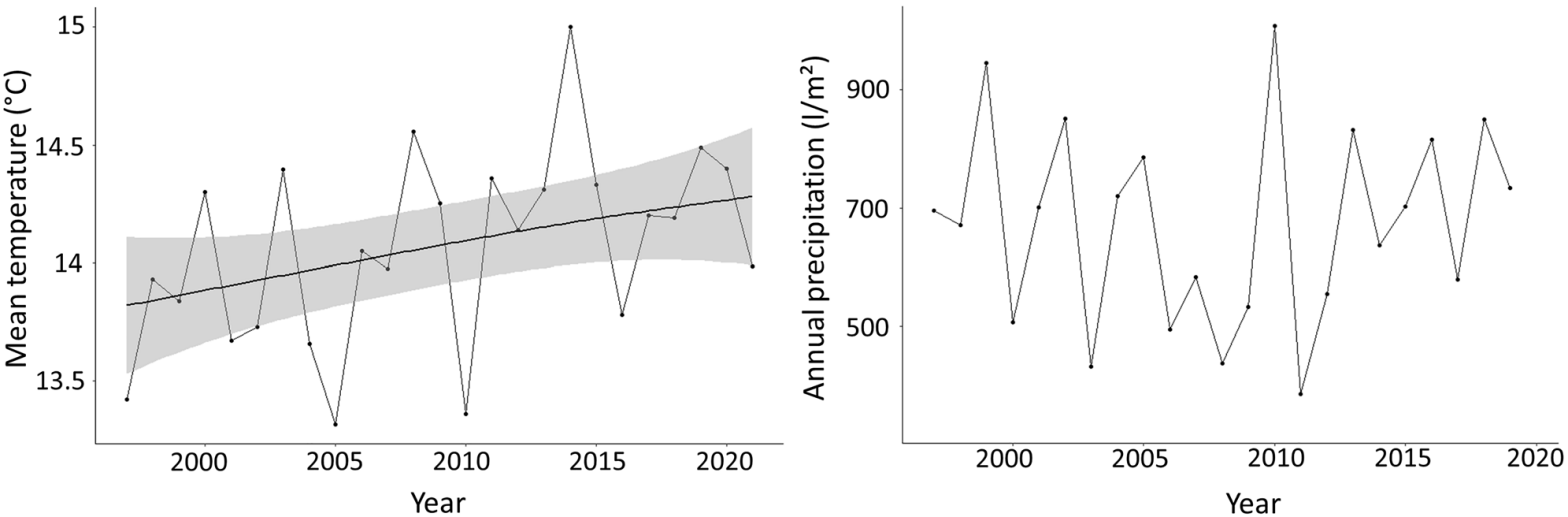
Changes in mean annual temperature (left) and precipitation (right) from 1997 to 2020 (sampling period) in the study region. The significantly increasing trend in mean annual temperature (as suggested by a Mann–Kendall test) is indicated by the black line (the grey shading indicates the confidence interval). In annual precipitation, no trend could be found.

The Mann–Kendall test for the mean annual temperature in the study region indicated a significant trend of increasing temperatures (*p* = 0.03), while there was no change in annual total precipitation (*p* = 0.94) in the last 20 years (Fig. 2). On average, the temperature increased by 0.57 °C from 1997 to 2020.

**Figure 3:**
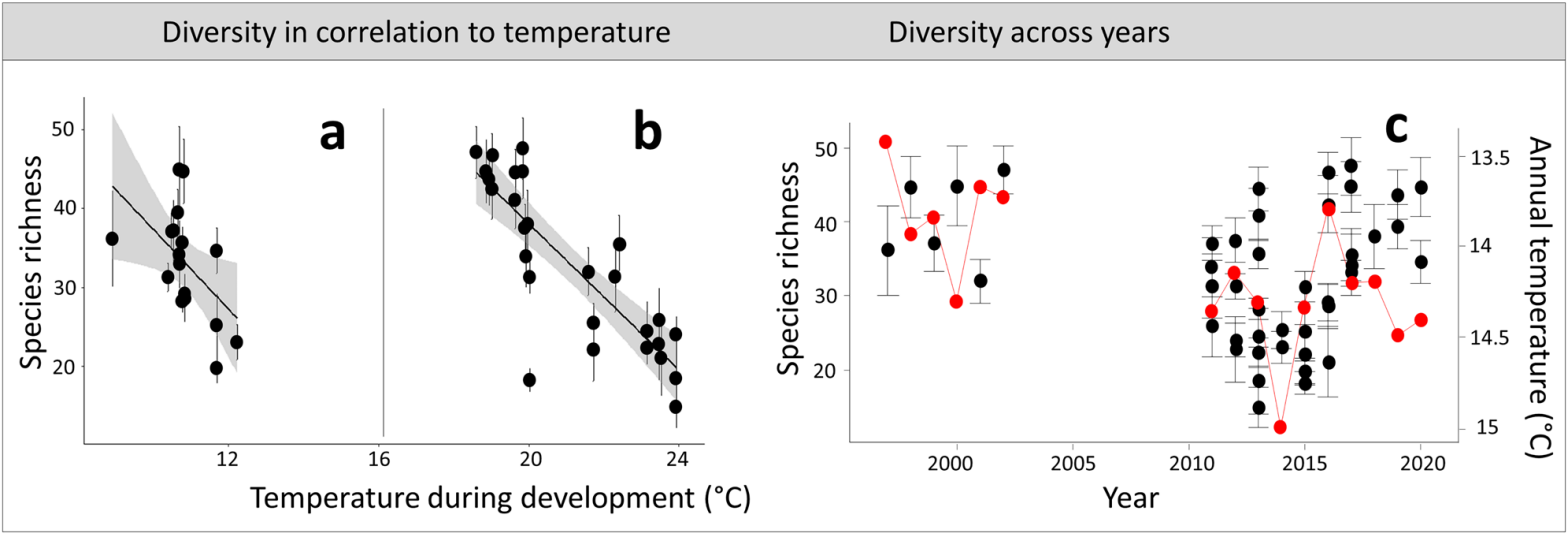
Changes in species richness in relation to temperature. On the left, standardized species richness in relation to T_Larv_. The linear models tested (see Table 1) are indicated by the black line (separately for overwintering larvae (**a**) and summer-developing larvae (**b**)). On the right, standardized species diversity from 1997 to 2020 (**c**). The red line indicates the annual temperature in these years and shows how inter-annual species diversity fluctuations might be explainable by annual temperature.

Concerning our first hypothesis, moth diversity was significantly correlated with T_Larv_, with higher temperatures resulting in lower species richness (Fig. 3). Observed inter-annual variation in species richness was well explained by climate variation (Fig. 3). Both, species richness of species with overwintering larvae and of species with summer-developing larvae were negatively affected by increasing temperatures. The effect was more pronounced for summer larvae, confirming our first hypothesis (Table 1). Contrary to our expectations, H_Larv_ did not affect species richness (Table 1).

**Table 1:** Results of the linear mixed-effects models for species richness of species with summer-developing and overwintering larvae (Fig. 1), including either the mean temperature during larval development (T_Larv_) or the monthly precipitation rates during larval development (H_Larv_) as predictors.

Response	Predictor	Samplesize	β-coefficient	*p*-value	R^2^ (marginal/conditional)
Overwintering Larvae	T_Larv_	17	−3.65	0.09	0.26/0.26
	H_Larv_	17	0.07	0.97	< 0.001/ < 0.001
Summer-Developing larvae	**T**_**Larv**_	**27**	**−8.72**	** < 0.001**	**0.67/0.69**
	H_Larv_	26	2.03	0.58	0.04/0.04

*P*-values are adjusted for multiple comparisons by False discovery rate control. Significant results are marked in bold.

**Figure 4:**
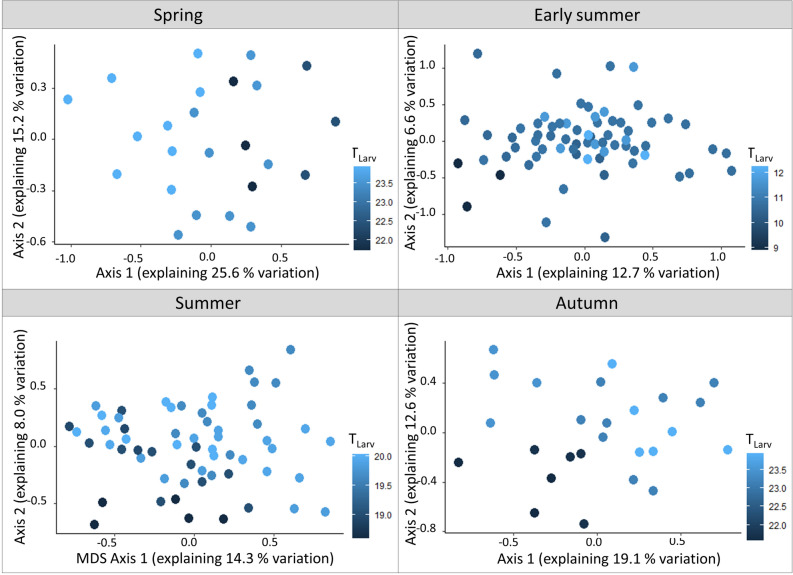
Non-metric multidimensional scaling (NMDS) ordination of the spring, early summer, summer and autumn moth communities, based on Sørensen distance. The color gradient indicates T_Larv_ (in °C) with light blue indicating warm temperatures and dark blue indicating cool temperatures.

**Figure 5:**
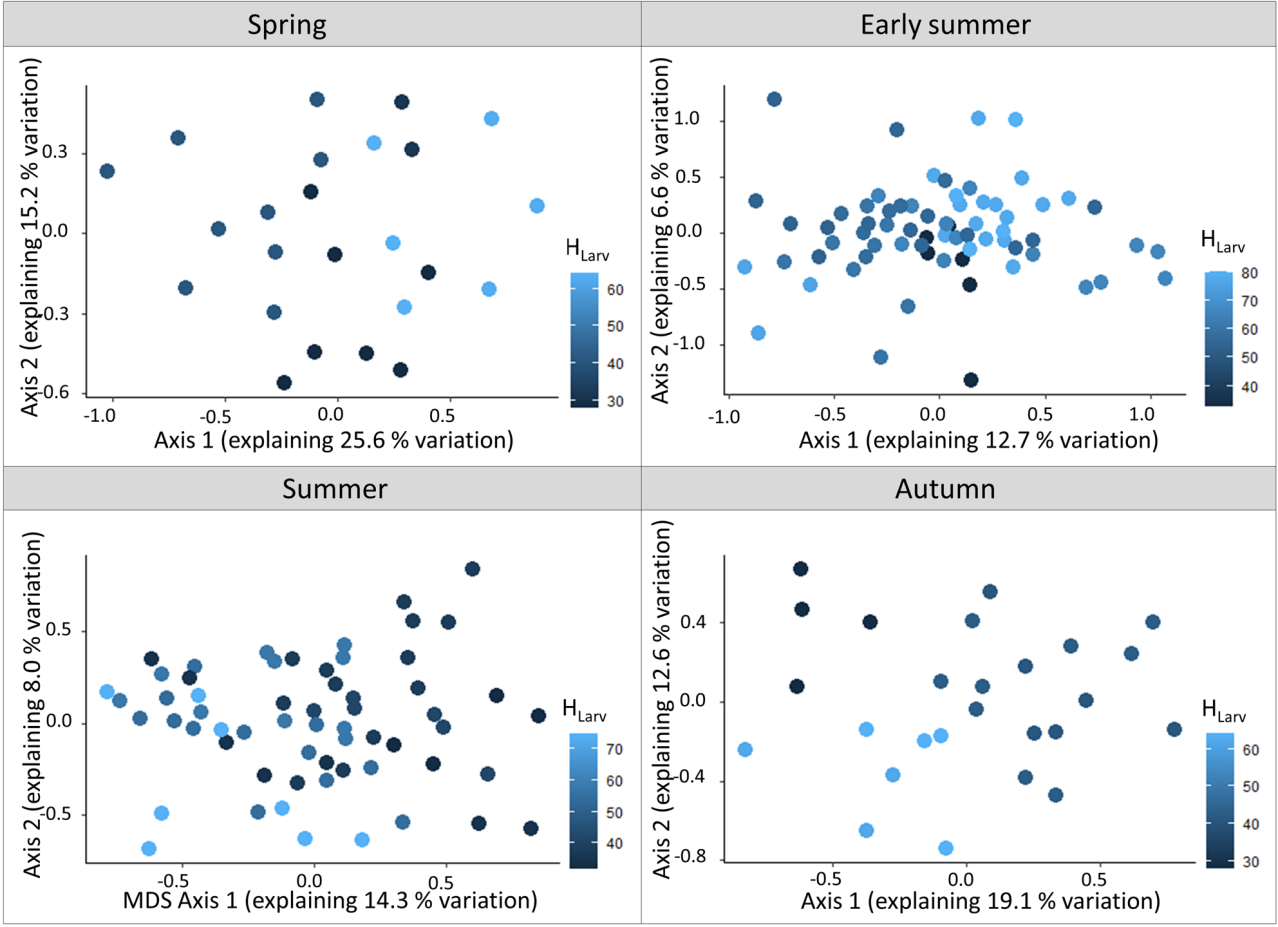
Non-metric multidimensional scaling (NMDS) ordination of the spring, early summer, summer and autumn moth communities, based on Sørensen distance. The color gradient indicates H_Larv_ (in l/m^2^) with light blue indicating high mean monthly precipitation rates and dark blue indicating low mean monthly precipitation rates.

Concerning our second hypothesis, T_Larv_ and H_Larv_ had a significant influence on moth community composition. The community composition of summer-developing larvae (especially spring and autumn moths) was strongly dependent on T_Larv_ and H_Larv_ (explaining up to 24% in variation). Yet, summer moth communities with larvae developing in early summer were less affected. Communities with overwintering larvae (i.e. early summer moths) were least affected by climate (explaining less than 10% of variation) (Table 2, Figs. 4, 5).

**Table 2:** Results of the permutation tests (based of 999 permutations). Given are the R^2^ and the p-values for the effects of T_Larv_ and H_Larv_ on sampled moth communities as well as for the trap type used (automated vs. manual light trap).

Moth community	T_Larv_		H_Larv_		Trap type	
	R^2^	*p*-value	R^2^	*p*-value	R^2^	*p*-value
Spring (summer-developing larvae)	0.14	0.001	0.10	0.001	0.07	0.04
Early summer (overwintering larvae)	0.04	0.001	0.03	0.004	0.06	0.001
Summer (early summer-developing larvae)	0.05	0.001	0.05	0.001	0.07	0.001
Autumn (summer-developing larvae)	0.11	0.001	0.13	0.001	0.07	0.006

The calculations were based on Sørensen dissimilarity.

## Discussion

We found changes in species diversity in relation to T_Larv_ and H_Larv_ in PsV throughout the last 20 years. Significant correlations between species richness and T_Larv_ indicate that macroclimate is an important determinant for population fluctuations. However, over the last 20 years, communities seem still resilient to annual temperature fluctuations (i.e. species diversity increases in cooler years and we observed no legacy effect of warm summers on subsequent species richness, see Fig. 3). However, with a trend of increasing mean temperatures in the study region, it is likely that hot summers will become more severe and frequent in the future^37,38^, which might possibly decrease community resilience. Potential moth diversity loss might have cascading effects on important ecosystem services and food webs, reducing nocturnal pollination and – in terms of co-occurring reduced biomass—the amount of available food e.g., for birds and bats^39,40^.

Focusing on α-diversity, the strength of response between species richness and T_Larv_ was more pronounced for species with summer-developing larvae than for species with overwintering larvae. This observation is in line with our expectation (Hypothesis 1) and seems to confirm that especially in the summer months high temperatures and drought pose a challenge to larvae. Overwintering larvae, in contrast, might be less affected as temperatures during the autumn and spring months are still moderate^2^, and higher precipitation rates result in high food plant quality. However, this finding likely only holds true for warm-adapted and Mediterranean species adapted to mild winters. For cold-adapted species with overwintering larvae, in contrast, increasing temperatures repeatedly have been found to increase mortality^41^. Cold-adapted species are able to endure longer frost periods in winter, yet, they are sensitive to mild and moist winters. As an example, *Boloria eunomia* and *Arctia caja* are prone to diseases and fungal infections, when winters become too mild^41,42^.

H_Larv_ was not a significant predictor for α-diversity. Monthly precipitation might not represent a sufficiently accurate proxy for water availability. A daily precipitation concentration index (i.e. the temporal distribution of rain) might be more appropriate, as the regular supply of water might be more important for biotic communities than the mere amount of rain. In fact, the precipitation concentration index tends to increase in the Mediterranean region, indicating that rain events will occur more irregularly in the future^43^. Integrating the combined effects of the rain amount and the precipitation concentration index as predictors for species diversity should, therefore, be the next step to evaluate climate change effects on insect communities.

T_Larv_ and H_Larv_ were both significant predictors for the community composition. The fact that H_Larv_ was a significant predictor for the community composition but not for species α-diversity might hint that mere species α-diversity analyses might fail to capture fine-nuanced diversity changes, especially when the predictors are coarse^44^. Yet, also here, a precipitation concentration index might be a more appropriate predictor (see above). As expected, the effect was most pronounced in communities with summer-developing larvae, supporting our second hypothesis. Especially spring and autumn moth communities were defined as communities with summer-developing larvae (Fig. 1). Hot summers, therefore, affect the most sensitive developmental stage of these communities. For summer moth communities, with their larvae developing in early summer, the effects of T_Larv_ and H_Larv_ were less pronounced. Summer moth communities might be less affected by climate change, as the larvae hatch in spring when the conditions are moderate. The first larval instars of summer moths, therefore, might still be unaffected by heat and drought.

Finally, adult species that normally occur during the summertime, might increasingly also be found in spring and autumn flight periods, due to phenological shifts^45^, prolonged egg and pupal stages^21^, summer dormancy^20^, or the occurrence of multiple generations across the year, prolonging their flight period to the spring and autumn time^46^. As a consequence, especially the composition of the spring and autumn communities changes, while community composition shifts in early summer and summer moth assemblages are much less pronounced (although significant).

One question remains: Does climate change affect larval development directly due to exceeded temperature optima, or is it an indirect effect of reduced food plant quality, that leads to decreasing species richness after hot summers? Concerning direct effects, laboratory experiments on model organisms can give some hints of how temperature affects larval development. Experiments on *Spodoptera frugiperda* and *Helicoverpa armigera* resulted in rather linear correlations, indicating that larvae develop fastest at high temperatures^47,48^. Yet, fast development does not equal high survival rates, which have been found to peak at temperatures of about 20 °C in many species^49,50^. The highest survival rate temperature of these model organisms, therefore, matches the summer temperature with the highest species richness in our field survey, while warmer temperatures resulted in increased mortality. Exceeded temperature optima therefore might not only be a problem for cold-adapted and tropical species but also Mediterranean and temperate species^51^.

Indirect effects of climate change might occur when lower food plant quality impacts the larval development of herbivorous insects. Yet, the interplay between dietary quality and larval fitness is not completely understood. Some species benefit from reduced plant health, as defense mechanisms in weakened plants are turned down (following the plant stress hypothesis of White^52^). However, reduced water content and lower nutritional value can also negatively affect a caterpillar’s survival (following the plant vigor hypothesis of Price^53^). Both hypotheses—although seemingly contradictory – represent two sides of the complex interplay between food plants and herbivores. Finally, the best quality food plant might be somewhere in between both hypotheses and also depend on the species preferences^54^. With our field case study, we cannot disentangle if species are threatened by direct climate change effects, such as exceeded temperature optima, or indirect effects, such as reduced food plant quality. Here, laboratory experiments are urgently needed, to get a mechanistic understanding of how climate change affects biotic communities.

## Conclusions

Correlations between species diversity and climate during larval development suggest that Mediterranean moth communities are prone to increased summer heat and drought as predicted by climate change scenarios, possibly leading to local extinctions. Particularly species with summer-developing larvae might suffer due to exceeded temperature optima and low food plant quality. Changes in insect diversity caused by climate change might not be relevant in the Mediterranean area only, where summer heat might exceed the species’ physiological optima but also in Central Europe where optima will likely be exceeded in the future. Further, our study suggests that climate change is increasingly becoming a threat not only for cold-adapted species^42^ but also for thermophilous insects in Mediterranean climate zones.

## Supplementary Information

Supplementary Information 1.

Supplementary Information 2.

## Data Availability

All data, including raw species lists and temperature data, are available as supplementary material to this manuscript.
