# Supplementary material for: Mediterranean moth diversity is sensitive to increasing temperatures and drought under climate change
Source: Sci Rep. 2022 Aug 25;12:14473. doi: 10.1038/s41598-022-18770-z (PMC9411567; doi:10.1038/s41598-022-18770-z)
Supplement: Supplementary file 1 — Supplementary Information 1. [file 41598_2022_18770_MOESM1_ESM.docx]

**Supplement**

Table S1: List of all the samples, sampling dates, sampling locations, and additional comments. The column on the left indicates which samples were pooled for species interpolation-extrapolation, building one data point (and confidence intervals) in Figure 3.

| **pooled together** | **year** | **traptype** | **season** | **date** | **location** | **comment** |
| --- | --- | --- | --- | --- | --- | --- |
|  | 1997 | manual | early summer | 26.05.1997 | Ca Vecchia | Hereafter abbreviated CV |
|  | 1997 | manual | early summer | 27.05.1997 | CV |  |
|  | 1997 | manual | early summer | 29.05.1997 | CV |  |
|  | 1998 | manual | early summer | 02.06.1998 | Ca Vecchia (Forest) | Hereafter abbreviated: CV(F) |
|  | 1998 | manual | early summer | 05.06.1998 | CV |  |
|  | 1998 | manual | early summer | 09.06.1998 | CV |  |
|  | 1998 | manual | early summer | 11.06.1998 | CV |  |
|  | 1998 | manual | early summer | 30.05.1998 | CV |  |
|  | 1999 | manual | early summer | 03.06.1999 | CV |  |
|  | 1999 | manual | early summer | 23.05.1999 | CV |  |
|  | 1999 | manual | early summer | 26.05.1999 | CV |  |
|  | 1999 | manual | early summer | 27.05.1999 | CV |  |
|  | 1999 | manual | early summer | 31.05.1999 | CV |  |
|  | 2000 | manual | early summer | 14.06.2000 | CV |  |
|  | 2000 | manual | early summer | 16.06.2000 | Ca Vecchia (South) | Hereafter bbreviated: CV(S) |
|  | 2000 | manual | early summer | 19.06.2000 | CV(S) |  |
|  | 2000 | manual | early summer | 20.06.2000 | CV(F) |  |
|  | 2000 | manual | early summer | 21.06.2000 | CV |  |
|  | 2001 | manual | fall | 01.09.2001 | CV |  |
|  | 2001 | manual | fall | 02.09.2001 | CV |  |
|  | 2001 | manual | fall | 03.09.2001 | CV |  |
|  | 2001 | manual | fall | 04.09.2001 | V7 |  |
|  | 2002 | manual | summer | 02.07.2002 | Ca Fossatone | Hereafter abbreviated: CF |
|  | 2002 | manual | summer | 09.07.2002 | CV |  |
|  | 2002 | manual | summer | 17.07.2002 | CF |  |
|  | 2002 | manual | summer | 21.07.2002 | PsV North | Hereafter abbreviated: PsV(N) |
|  | 2002 | manual | summer | 23.07.2002 | CV |  |
|  | 2002 | manual | summer | 23.07.2002 | PSV(N) |  |
|  | 2011 | automated | fall |  | open grassland | Consists of PsV(N), CV(S), G1, G2 |
|  | 2011 | automated | fall |  | hygrophil forest | Consists of V1, V7, Hy1, V23 |
|  | 2011 | automated | fall |  | oak forest | Consists of V4, V8, Oa1, V15 |
|  | 2011 | automated | fall |  | Reed | Consists of R1-4 |
|  | 2011 | automated | early summer |  | oak forest | Consists of V4, V8, Oa1, V15 |
|  | 2011 | automated | early summer |  | Reed | Consists of R 1-4 |
|  | 2011 | automated | early summer |  | hygrophil forest | Consists of V1, V7, Hy1, V23 |
|  | 2011 | automated | early summer |  | open grassland | Consists of PsV(N), CV(S), G1, G2 |
|  | 2011 | automated | summer |  | Oak forest | Consists of V4, V8, Oa1, V15 |
|  | 2011 | automated | summer |  | reed | Consists of R 1-4 |
|  | 2011 | automated | summer |  | hygrophil forest | Consists of V1, V7, Hy1, V23 |
|  | 2011 | automated | summer |  | open grassland | Consists of PsV(N), CV(S), G1, G2 |
|  | 2011 | automated | spring |  | Oak forest | Consists of V4, V8, Oa1, V15 |
|  | 2011 | automated | spring |  | reed | Consists of R 1-4 |
|  | 2011 | automated | spring |  | hygrophil forest | Consists of V1, V7, Hy1, V23 |
|  | 2011 | automated | spring |  | open grassland | Consists of PsV(N), CV(S), G1, G2 |
|  | 2012 | manual | spring | 18.04.2012 | CV(F) |  |
|  | 2012 | manual | spring | 19.04.2012 | V6 |  |
|  | 2012 | manual | spring | 20.04.2012 | CV(F) |  |
|  | 2012 | manual | spring | 21.04.2012 | CV |  |
|  | 2012 | manual | spring | 23.04.2012 | Ca Nova | Hereafter abbreviated: CN |
|  | 2012 | manual | spring | 24.04.2012 | V16 |  |
|  | 2012 | manual | spring | 25.04.2012 | CV |  |
|  | 2012 | manual | early summer | 03.06.2012 | CV |  |
|  | 2012 | manual | early summer | 05.06.2012 | CV(F) |  |
|  | 2012 | manual | early summer | 06.06.2012 | CN |  |
|  | 2012 | manual | early summer | 09.06.2012 | PSV(N) |  |
|  | 2012 | manual | early summer | 12.06.2012 | CV(F) |  |
|  | 2012 | manual | early summer | 14.06.2012 | Ca Vecchia (Reed) | Hereafter abbreviated: CV(R) |
|  | 2012 | manual | summer | 05.08.2012 | CV(F) |  |
|  | 2012 | manual | summer | 07.08.2012 | PSV(N) |  |
|  | 2012 | manual | summer | 08.08.2012 | CN |  |
|  | 2012 | manual | summer | 10.08.2012 | CV(R) |  |
|  | 2012 | manual | summer | 13.08.2012 | CV |  |
|  | 2012 | manual | summer | 15.08.2012 | Canal (Ca) |  |
|  | 2012 | manual | fall | 02.10.2012 | CN |  |
|  | 2012 | manual | fall | 03.10.2012 | CV |  |
|  | 2012 | manual | fall | 05.10.2012 | PSV(N) |  |
|  | 2012 | manual | fall | 07.10.2012 | R2 |  |
|  | 2012 | manual | fall | 08.10.2012 | CV(F) |  |
|  | 2012 | manual | fall | 10.10.2012 | V24 |  |
|  | 2013 | manual | spring | 15.04.2013 | CV |  |
|  | 2013 | manual | spring | 18.04.2013 | CN |  |
|  | 2013 | manual | spring | 23.04.2013 | PSV North |  |
|  | 2013 | manual | early summer | 09.06.2013 | CV(F) |  |
|  | 2013 | manual | early summer | 11.06.2013 | CN |  |
|  | 2013 | manual | early summer | 14.06.2013 | V1 |  |
|  | 2013 | manual | early summer | 16.06.2013 | CV |  |
|  | 2013 | manual | early summer | 19.06.2013 | PSV(N) |  |
|  | 2013 | manual | early summer | 21.06.2013 | V26 |  |
|  | 2013 | manual | summer | 05.08.2013 | CV |  |
|  | 2013 | manual | summer | 08.08.2013 | CN |  |
|  | 2013 | manual | summer | 10.08.2013 | CV(R) |  |
|  | 2013 | manual | summer | 12.08.2013 | CV(F) |  |
|  | 2013 | manual | summer | 15.08.2013 | V1 |  |
|  | 2013 | manual | fall | 03.10.2013 | CN |  |
|  | 2013 | manual | fall | 09.10.2013 | CV |  |
|  | 2013 | manual | fall | 10.10.2013 | PSV(N) |  |
|  | 2013 | automated | spring | 15.04.2013 |  | Consists of V25, V27, V28, V30 |
|  | 2013 | automated | spring | 16.04.2013 |  | Consists of PSV(N), V2, V3, V4 |
|  | 2013 | automated | spring | 18.04.2013 |  | Consists of V13, V18, V19, V21 |
|  | 2013 | automated | spring | 19.04.2013 |  | Consists of V5, V8, V9, V14 |
|  | 2013 | automated | spring | 23.04.2013 |  | Consists of V25, V27, V28, V30 |
|  | 2013 | automated | spring | 24.04.2013 |  | Consists of PSV(N), V2, V3, V4 |
|  | 2013 | automated | early summer | 10.06.2013 |  | Consists of V25, V27, V28, V30 |
|  | 2013 | automated | early summer | 11.06.2013 |  | Consists of PSV(N), V2, V3, V4 |
|  | 2013 | automated | early summer | 13.06.2013 |  | Consists of V13, V18, V19, V21 |
|  | 2013 | automated | early summer | 14.06.2013 |  | Consists of V5, V8, V9, V14 |
|  | 2013 | automated | early summer | 15.06.2013 |  | Consists of V25, V27, V28, V30 |
|  | 2013 | automated | early summer | 16.06.2013 |  | Consists of PSV(N), V2, V3, V4 |
|  | 2013 | automated | early summer | 18.06.2013 |  | Consists of V13, V18, V19, V21 |
|  | 2013 | automated | early summer | 19.06.2013 |  | Consists of V5, V8, V9, V14 |
|  | 2013 | automated | summer | 05.08.2013 |  | Consists of V25, V27, V28, V30 |
|  | 2013 | automated | summer | 06.08.2013 |  | Consists of PSV(N), V2, V3, V4 |
|  | 2013 | automated | summer | 08.08.2013 |  | Consists of V13, V18, V19, V21 |
|  | 2013 | automated | summer | 09.08.2013 | V14 | Only one sample due to bad weather |
|  | 2013 | automated | summer | 10.08.2013 |  | Consists of V5, V8, V9, |
|  | 2013 | automated | summer | 11.08.2013 |  | Consists of V25, V27, V28, V30 |
|  | 2013 | automated | summer | 12.08.2013 |  | Consists of V2, V3, V4 |
|  | 2013 | automated | summer | 15.08.2013 | PSV(N) | Only one sample due to bad weather |
|  | 2013 | automated | fall | 30.09.2013 |  | Consists of V25, V27, V28, V30 |
|  | 2013 | automated | fall | 01.10.2013 |  | Consists of PSV(N), V2, V3, V4 |
|  | 2013 | automated | fall | 03.10.2013 |  | Consists of V13, V18, V19, V21 |
|  | 2013 | automated | fall | 04.10.2013 |  | Consists of V5, V8, V9, V14 |
|  | 2013 | automated | fall | 09.10.2013 |  | Consists of PSV(N), V2, V3, V4 |
|  | 2013 | automated | fall | 10.10.2013 |  | Consists of V25, V27, V28, V30 |
|  | 2014 | manual | early summer | 05.06.2014 | CV |  |
|  | 2014 | manual | early summer | 09.06.2014 | CN |  |
|  | 2014 | manual | early summer | 12.06.2014 | PSV(N) |  |
|  | 2014 | manual | fall | 23.09.2014 | CV |  |
|  | 2014 | manual | fall | 25.09.2014 | CN |  |
|  | 2014 | manual | fall | 27.09.2014 | PSV(N) |  |
|  | 2014 | manual | fall | 30.09.2014 | CV(R) |  |
|  | 2015 | manual | spring | 20.04.2015 | CN |  |
|  | 2015 | manual | spring | 22.04.2015 | PSV(N) |  |
|  | 2015 | manual | spring | 24.04.2015 | CV |  |
|  | 2015 | manual | early summer | 01.06.2015 | CV |  |
|  | 2015 | manual | early summer | 03.06.2015 | CN |  |
|  | 2015 | manual | early summer | 09.06.2015 | PSV(N) |  |
|  | 2015 | manual | summer | 27.07.2015 | CN |  |
|  | 2015 | manual | summer | 29.07.2015 | CV |  |
|  | 2015 | manual | summer | 03.08.2015 | PSV(N) |  |
|  | 2015 | manual | summer | 01.09.2015 | CV |  |
|  | 2015 | manual | summer | 06.09.2015 | CN |  |
|  | 2015 | manual | summer | 12.09.2015 | PSV(N) |  |
|  | 2015 | automated | early summer | 01.06.2015 |  | Consists of V19, V23, V27 |
|  | 2015 | automated | early summer | 06.06.2015 | V8 | only one sample due to technical problems |
|  | 2015 | automated | summer | 27.07.2015 |  | Consists of V1, V2, V8 |
|  | 2015 | automated | summer | 03.08.2015 |  | Consists of V9, V10, V21 |
|  | 2015 | automated | summer | 06.08.2015 | V19 | only one sample due to technical problems |
|  | 2015 | automated | summer | 30.07.2015 |  | Consists of V23, V25, V27 |
|  | 2016 | manual | spring | 18.04.2016 | CV |  |
|  | 2016 | manual | spring | 21.04.2016 | PSV(N) |  |
|  | 2016 | manual | early summer | 31.05.2016 | CV |  |
|  | 2016 | manual | early summer | 07.06.2016 | PSV(N) |  |
|  | 2016 | manual | summer | 25.07.2016 | CN |  |
|  | 2016 | manual | summer | 29.07.2016 | PSV(N) |  |
|  | 2016 | manual | summer | 31.07.2016 | CV |  |
|  | 2016 | manual | summer | 30.08.2016 | CV |  |
|  | 2016 | manual | summer | 03.09.2016 | CN |  |
|  | 2016 | manual | summer | 11.09.2016 | PSV(N) |  |
|  | 2016 | automated | early summer | 29.05.2016 |  | Consists of V4, V5 |
|  | 2016 | automated | early summer | 08.06.2016 |  | Consists of V7, V11 |
|  | 2016 | automated | early summer | 03.06.2016 |  | Consists of V16, V17 |
|  | 2016 | automated | early summer | 31.05.2016 |  | Consists of V24, V26, V30 |
|  | 2016 | automated | summer | 23.07.2016 |  | Consists of V4, V17 |
|  | 2016 | automated | summer | 29.07.2016 |  | Consists of V5, V11, V16 |
|  | 2016 | automated | summer | 31.07.2016 |  | Consists of V20, V26 |
|  | 2016 | automated | summer | 25.07.2016 |  | Consists of V24, V30 |
|  | 2017 | manual | spring | 22.04.2017 | CV |  |
|  | 2017 | manual | spring | 24.04.2017 | PSV(N) |  |
|  | 2017 | manual | spring | 25.04.2017 | CN |  |
|  | 2017 | manual | early summer | 13.06.2017 | PSV(N) |  |
|  | 2017 | manual | early summer | 17.07.2017 | CN |  |
|  | 2017 | manual | summer | 29.08.2017 | CV |  |
|  | 2017 | manual | summer | 31.08.2017 | CN |  |
|  | 2017 | automated | early summer | 27.05.2017 |  | Consists of V1, V2, V3 |
|  | 2017 | automated | early summer | 05.06.2017 |  | Consists of V6, V9, V10 |
|  | 2017 | automated | early summer | 01.06.2017 |  | Consists of V12, V14, V22 |
|  | 2017 | automated | early summer | 13.06.2017 |  | Consists of V13, V20. |
|  | 2017 | automated | early summer | 31.05.2017 |  | Consists of V15, V18, |
|  | 2017 | automated | early summer | 15.06.2017 | V21 |  |
|  | 2017 | automated | early summer | 29.05.2017 |  | Consists of V25, V28, V29 |
|  | 2017 | automated | summer | 24.07.2017 |  | Consists of V3, V6, V7 |
|  | 2017 | automated | summer | 20.07.2017 |  | Consists of V12, V13, V14 |
|  | 2017 | automated | summer | 21.07.2017 |  | Consists of V15, V18, |
|  | 2017 | automated | summer | 22.07.2017 |  | Consists of V22, V28, |
|  | 2017 | automated | summer | 17.07.2017 | V29 |  |
|  | 2018 | manual | summer | 28.08.2018 | CV |  |
|  | 2018 | manual | summer | 05.09.2018 | CN |  |
|  | 2019 | manual | early summer | 31.05.2019 | CN |  |
|  | 2019 | manual | early summer | 01.06.2019 | CV |  |
|  | 2019 | manual | early summer | 02.06.2019 | PSV(N) |  |
|  | 2019 | manual | summer | 27.08.2019 | CV |  |
|  | 2019 | manual | summer | 31.08.2019 | PSV(N) |  |
|  | 2019 | manual | summer | 04.09.2019 | CN |  |
|  | 2020 | manual | early summer | 10.06.2020 | PSV(N) |  |
|  | 2020 | manual | early summer | 11.06.2020 | CN |  |
|  | 2020 | manual | early summer | 12.06.2020 | CN |  |
|  | 2020 | manual | early summer | 14.06.2020 | CV |  |
|  | 2020 | manual | summer | 03.09.2020 | CN |  |
|  | 2020 | manual | summer | 04.09.2020 | PSV(N) |  |
|  | 2020 | manual | summer | 06.09.2020 | CV |  |

Table S2 (separate excel file): species abundance list with all sample nights (rows; Automated samples are pooled as described in the methods section). The first five columns give information on the predictors used: sampling year, trap type, season, T_Larv_, and H_Larv_.

Table S3: Coordinates for the weather stations used to gather precipitation rates

| Location | Latitude | Longitude |
| --- | --- | --- |
| Ravenna (city) | 44.40124 | 12.18363 |
| Ravenna (industrial area) | 44.46535 | 12.20555 |
| Marina di Ravenna | 44.49092 | 12.28387 |
| Classe | 44.37434 | 12.24073 |
| Fosso Ghiaia | 44.35337 | 12.28387 |
| Ponte Chiavica | 44.56128 | 12.24389 |
| San Pietro in Vincoli | 44.31392 | 12.16020 |

Table S4: Calculations based on A) only one trap type (manual) and B) only on one trap type and one location (CV). For the latter calculation, no samples were pooled. Each sampling night was used as independent sample.

| response A  (only manual trap) | predictor | samplesize | β-coefficient | p-value | R² |
| --- | --- | --- | --- | --- | --- |
| overwintering larvae | T_Larv_ | 12 | -3.76 | 0.10 | 0.24 |
|  | H_Larv_ | 12 | 0.60 | 0.81 | 0.006 |
| summer-developing larvae | **T_Larv_** | **18** | **-9.35** | **<0.001** | **0.83** |
|  | H_Larv_ | 17 | 3.47 | 0.18 | 0.12 |
| response B (same trap and location) | predictor | samplesize | β-coefficient | p-value | R² |
| overwintering larvae | T_Larv_ | 21 | -0.65 | 0.46 | 0.03 |
|  | H_Larv_ | 21 | 0.38 | 0.66 | 0.01 |
| summer-developing larvae | **T_Larv_** | **23** | **-3.00** | **<0.001** | **0.51** |
|  | H_Larv_ | 22 | 1.38 | 0.13 | 0.11 |


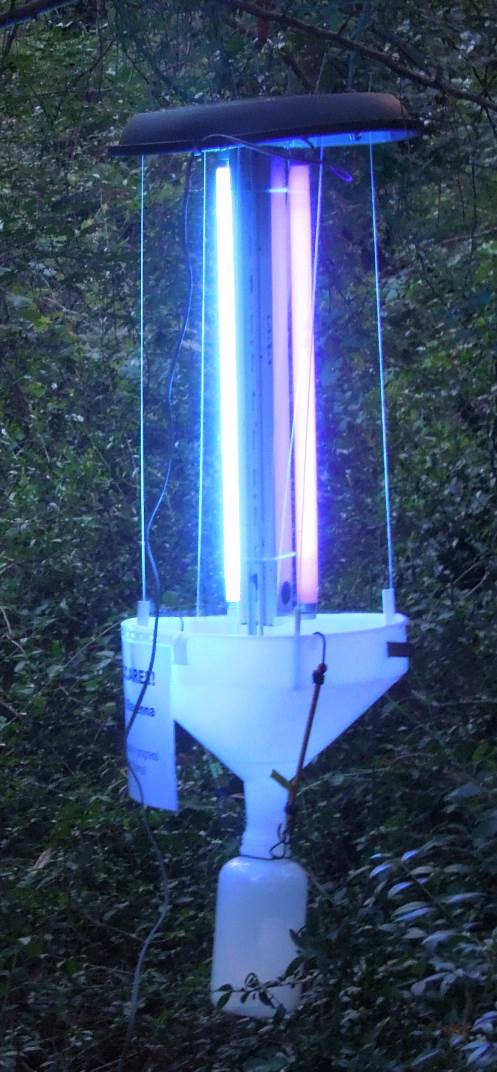

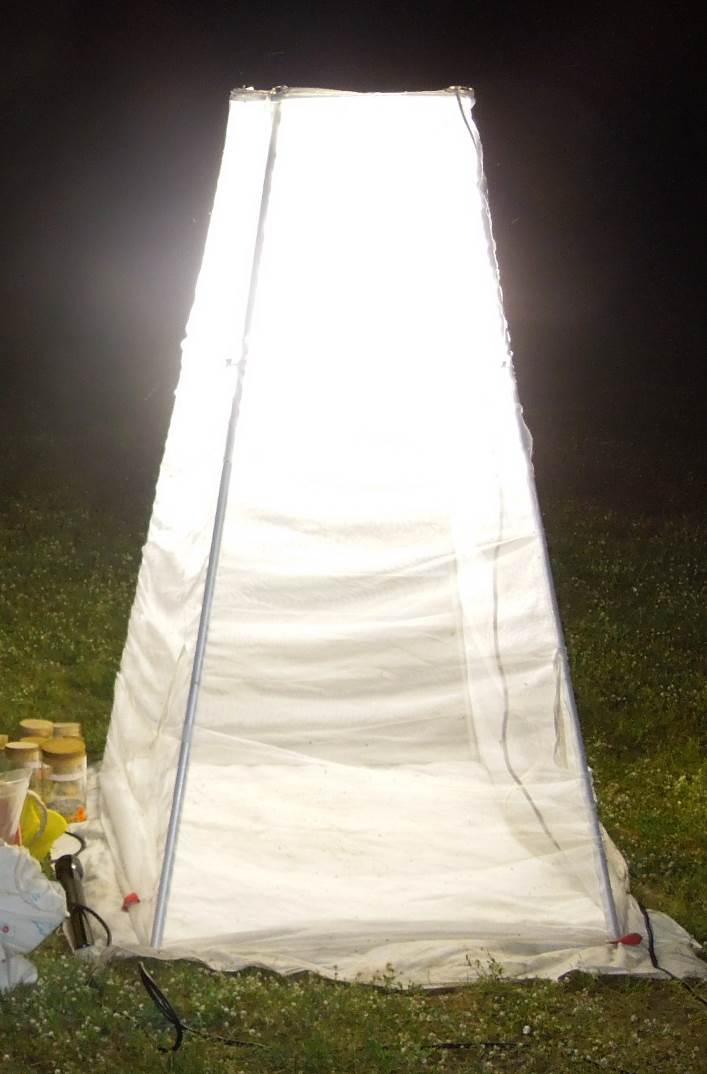


Fig. S1 Light trap types used. Left: Automated light trap, Right: manual light trap.


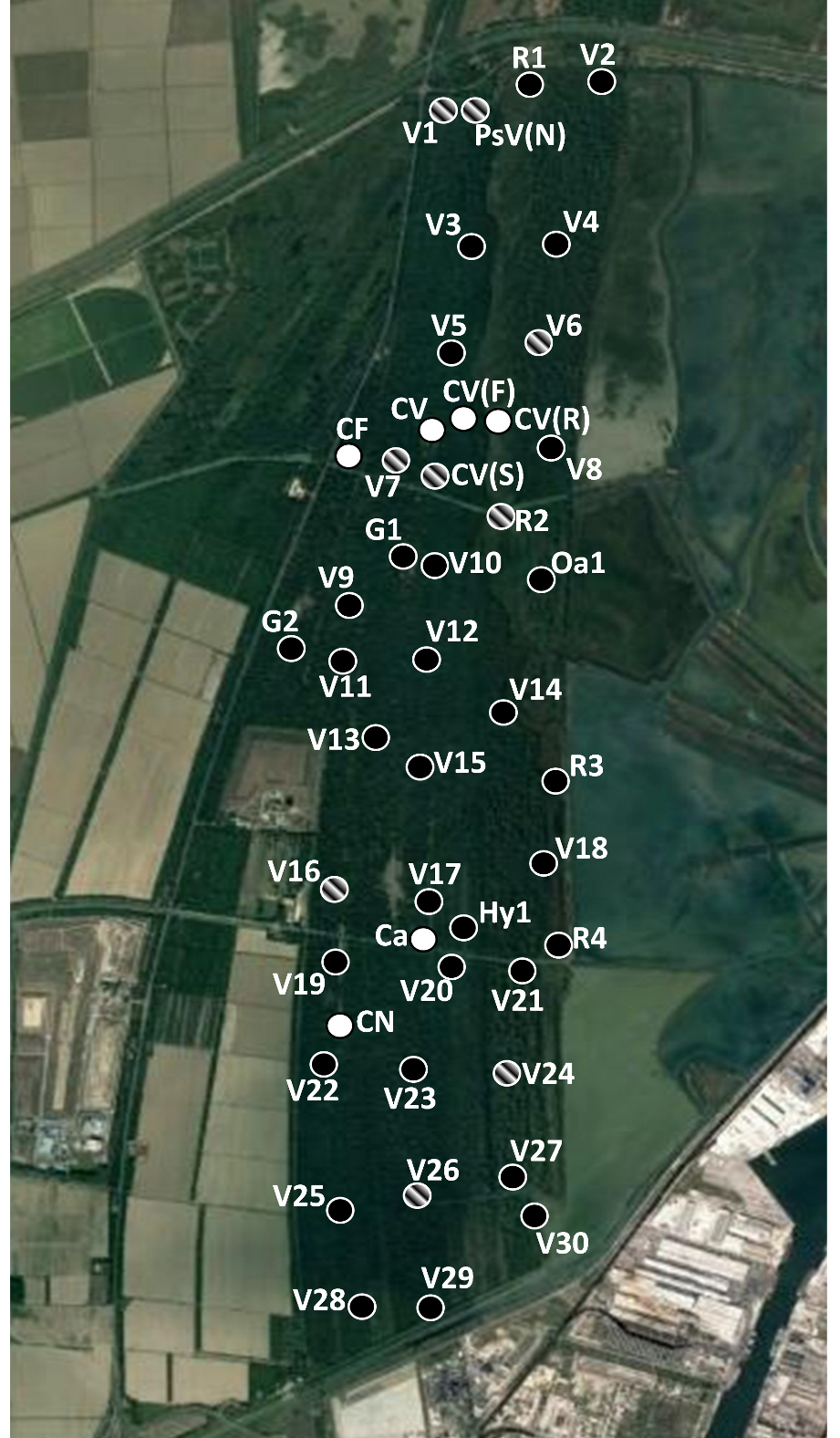


Fig. S2 map of the sampling locations. Black points indicate locations of automated light traps. White points indicate manual light trap locations. Striped locations were sampled by both trap types.


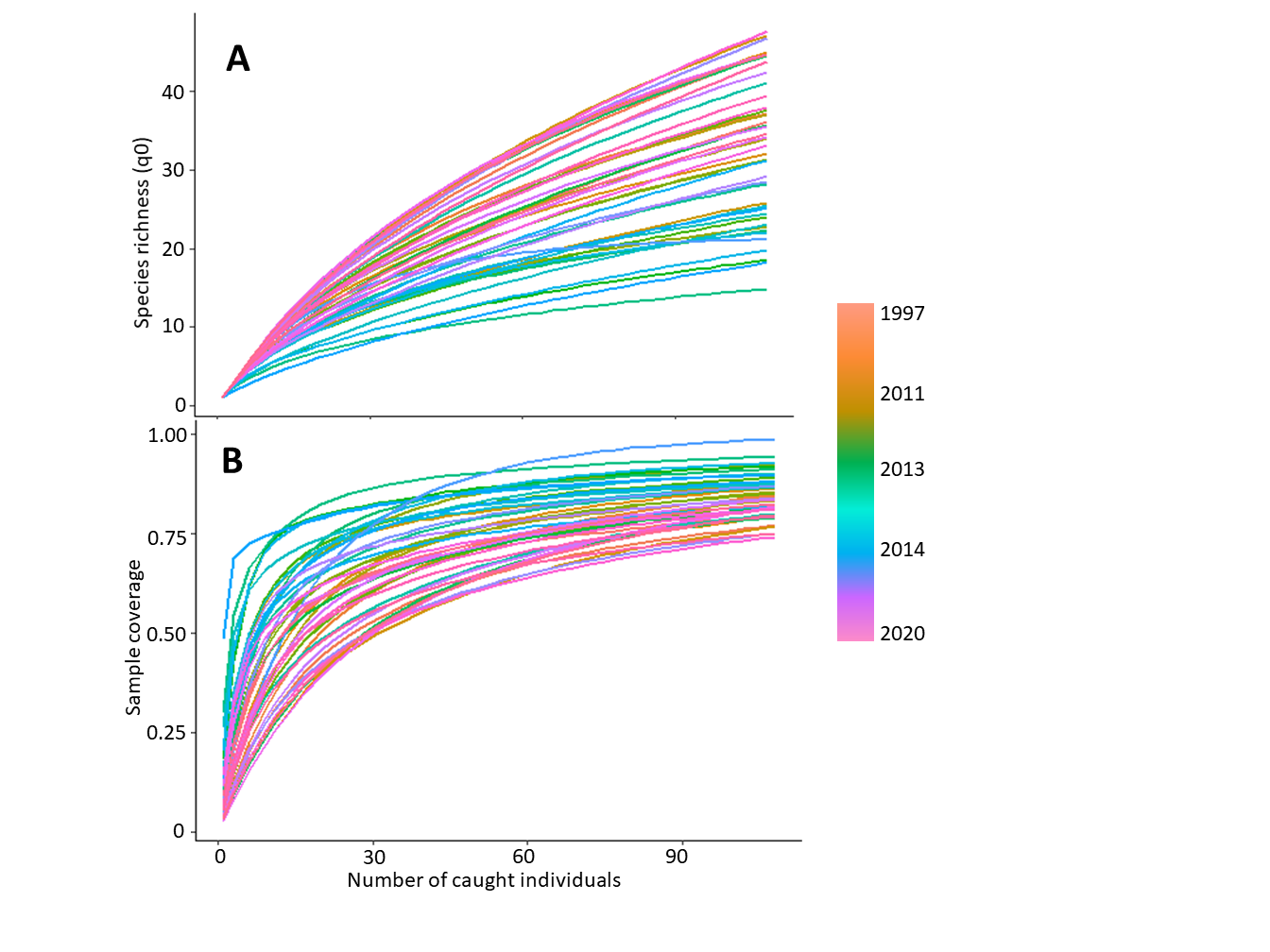


Fig. S3: Species interpolation-extrapolation (A) and sample coverage (B) of the available moth datasets. Seasons per year were treated separately, different trap types also were treated as separate datasets. All samples were interpolated to 108 individuals, except the smallest dataset (2016), which was extrapolated to the same number of individuals.
